# ABCB1 overexpression through locus amplification represents an actionable target to combat paclitaxel resistance in pancreatic cancer cells

**DOI:** 10.1186/s13046-023-02879-8

**Published:** 2024-01-02

**Authors:** Cecilia Bergonzini, Alessandro Gregori, Tessa M. S. Hagens, Vera E. van der Noord, Bob van de Water, Annelien J. M. Zweemer, Bircan Coban, Mjriam Capula, Giulia Mantini, Asia Botto, Francesco Finamore, Ingrid Garajova, Liam A. McDonnell, Thomas Schmidt, Elisa Giovannetti, Erik H. J. Danen

**Affiliations:** 1 Leiden Academic Center for Drug Research, Leiden University, Leiden, The Netherlands; 2 Physics of Life Processes, Leiden Institute of Physics, Leiden University, Leiden, The Netherlands; 3 Department of Medical Oncology, Cancer Center Amsterdam, Amsterdam UMC, VU University, Amsterdam, The Netherlands; 4 Cancer Pharmacology Lab, Fondazione Pisana Per La Scienza, San Giuliano, Pisa, Italy; 5 Proteomics and Metabolomics Lab, Fondazione Pisana Per La Scienza, San Giuliano, Pisa, Italy; 6 Department of Chemistry and Industrial Chemistry, University of Pisa, Pisa, Italy; 7 Medical Oncology Unit, University Hospital of Parma, Parma, Italy

**Keywords:** Pancreatic cancer, Paclitaxel resistance, ABCB1, Kinase-inhibitors

## Abstract

**Background:**

Pancreatic ductal adenocarcinoma (PDAC) is one of the deadliest types of cancer and the chemotherapies such as gemcitabine/nab-paclitaxel are confronted with intrinsic or acquired resistance. The aim of this study was to investigate mechanisms underlying paclitaxel resistance in PDAC and explore strategies to overcome it.

**Methods:**

Three paclitaxel (PR) and gemcitabine resistant (GR) PDAC models were established. Transcriptomics and proteomics were used to identify conserved mechanisms of drug resistance. Genetic and pharmacological approaches were used to overcome paclitaxel resistance.

**Results:**

Upregulation of ABCB1 through locus amplification was identified as a conserved feature unique to PR cells. ABCB1 was not affected in any of the GR models and no cross resistance was observed. The ABCB1 inhibitor verapamil or siRNA-mediated ABCB1 depletion sensitized PR cells to paclitaxel and prevented efflux of ABCB1 substrates in all models. ABCB1 expression was associated with a trend towards shorter survival in patients who had received gemcitabine/nab-paclitaxel treatment. A pharmacological screen identified known and novel kinase inhibitors that attenuate efflux of ABCB1 substrates and sensitize PR PDAC cells to paclitaxel.

**Conclusion:**

Upregulation of ABCB1 through locus amplification represents a novel, conserved mechanism of PDAC paclitaxel resistance. Kinase inhibitors identified in this study can be further (pre) clinically explored as therapeutic strategies to overcome paclitaxel resistance in PDAC.

**Supplementary Information:**

The online version contains supplementary material available at 10.1186/s13046-023-02879-8.

## Background

Pancreatic ductal adenocarcinoma (PDAC) is one of the most lethal cancers worldwide [1], with a 5-year overall-survival reached in only 10% of patients [2]. This poor prognosis is due to a lack of early biomarkers, limited therapeutic options, and inherent or acquired chemoresistance [3, 4]. Currently, surgical resection is the only curative option for patients diagnosed with PDAC but < 20% are diagnosed at an early stage and therefore eligible for surgery. In all other cases (i.e., advanced PDAC), chemotherapy using FOLFIRINOX or gemcitabine plus nab-paclitaxel are the only treatment options [5, 6]. Unfortunately, these chemotherapy regimens increase survival up to 13 months at most, largely due to development of chemoresistance. So far, immunotherapy has not been successful for PDAC patients and new therapies under investigation targeting the tumor or the tumor microenvironment have not reached the clinic [7–9]. Therefore, identifying strategies to combat PDAC resistance to currently used chemotherapies is of crucial importance.

Gemcitabine is a cytotoxic DNA-intercalating drug, which arrests aberrant cell proliferation. Chemoresistance to gemcitabine in PDAC has been extensively studied and reported to be multifactorial [10]. On the other hand, paclitaxel is a microtubule-stabilizing drug that impedes cell division leading to replication errors and cell death. Paclitaxel also potentiates gemcitabine efficacy by increasing intratumor uptake and inhibiting its inactivation by catabolizing enzymes [10, 11]. Mechanisms underlying paclitaxel resistance in PDAC are poorly understood [12, 13]. Three studies have investigated paclitaxel resistance in PDAC, reporting that it involves metabolic adaptation [14], sustained c-MYC activation [15], and expression of orexin receptor type 1 [16].

ATP-binding cassette (ABC) transporters are responsible for active transport of many substrates, including cytotoxic drugs, across the cell membrane towards the extracellular space. ABC transporters are therefore known as multidrug resistance pumps [13, 17]. The ABC family consists of 49 members, among which ABCB1 (also known as MDR1 or P-glycoprotein, P-gp) has been extensively studied in cancer. Overexpression of ABCB1 can mediate paclitaxel resistance in different tumor types, including colorectal, lung, ovarian and breast [18–21]. Surprisingly, in PDAC, ABC transporters, including ABCB1, have been associated with gemcitabine resistance [13, 22–26] but not with paclitaxel resistance.

In the present study, we have generated three independent paclitaxel- and gemcitabine resistant PDAC models and found that ABCB1 is amplified in paclitaxel resistant, but not in gemcitabine resistant PDAC cells. We show that pharmaceutical or genetic inhibition of ABCB1 effectively restores paclitaxel sensitivity in the resistant cell lines. We show that ABCB1 is expressed heterogeneously in PDAC patients. Moreover, as clinical trials of currently available ABCB1 inhibitors have not proven successful due to lack of efficacy or toxicity [21, 27], we screened a kinase-inhibitor (KI) library for KIs that attenuate efflux of ABCB1 substrates, thereby overcoming paclitaxel resistance. We identified several novel KIs that can be further (pre) clinically explored as therapeutic strategies in combination with paclitaxel to overcome paclitaxel resistance in PDAC and other cancers.

## Methods

### Materials and cell culture

Three PDAC cell lines were used: Patu-T (mesenchymal phenotype), kindly provided by Dr. Irma van Die (Amsterdam UMC, Amsterdam, The Netherland), Suit-2.028 (epithelial phenotype) and Suit-2.007 (mesenchymal phenotype), kindly provided by Dr. Adam Frampton (Imperial College London, London, UK). Patu-T were maintained in DMEM, supplemented with 10% heat-inactivated bovine fetal serum and 1% penicillin/streptomycin, while both Suit-2 cell lines were cultured in RPMI supplemented as described above. All cells were kept in humidified atmosphere of 5% CO_2_ and 95% air at 37 °C, subcultured twice a week, tested monthly for mycoplasma contamination by MycoAlert Mycoplasma Detection Kit (Westburg, Leusden, The Netherlands) and cell identity was verified by short tandem repeats (STR) profiling.

Gemcitabine was kindly provided by Eli Lilly Corporation (Indianapolis, IN, USA) and dissolved in sterile water. Paclitaxel and verapamil were obtained from Sigma (T7402 and V4629, Sigma-Aldrich, St. Luis, MO, USA).

### Generation of resistant cell lines

To establish gemcitabine-resistant (GR) and paclitaxel-resistant (PR) cell lines, concentrations causing 50% reduction in cell growth (IC_50_) were determined in parental cells. Cells were then exposed to the respective IC_50_ of the drug and grown for at least 2 weeks with the drug until reaching 80% confluency. After acquiring resistance, the drug concentration was doubled (2 × IC_50_) and cells were cultured until they could grow to confluence. The process was repeated with stepwise increasing drug concentrations until the maximum tolerated concentration was reached after 6–12 months. Parental cells never exposed to the drug were cultured in parallel with the resistant cells. To determine stable resistance, PR and GR cells were grown in drug-free medium and baseline growth and resistance to the maximum tolerated concentration was analyzed by SRB assay at regular intervals for up to 2 months. The resistance factor was calculated as the ratio of the IC_50_ of resistant versus IC_50_ of parental cells. For IC_50_ > 12 μM, the resistance factor was calculated using the maximum drug concentration used in the SRB assay. Batches of resistant cells used in experiments were maintained in drug-free medium ≤ 2 months.

### Immunohistochemistry

Expression of ABCB1 in PDAC patients was evaluated by immunohistochemistry (IHC) in paraffin-embedded tumor specimens from 32 PDAC patients who underwent resection and were treated with gemcitabine (1,000 mg/m^2^) plus nab-paclitaxel (125 mg/m^2^) as first-line therapy. All specimens were obtained after patient’s written consent approved by the Ethics Committee of “Area Vasta Emilia Nord” (protocol code 12003—17/03/2021). Tissue sections were stained overnight with rabbit anti-human ABCB1 (E1Y7S, mAb #13978; Cell Signaling Technology; dilution 1:400). Sections were reviewed independently by two researchers blinded to clinical data, who scored the immunostaining on the basis of staining intensities and number of stained cells as “low” or “high”. Overall survival (OS) was calculated from the date of pathologic diagnosis (i.e., the date of surgery/biopsy) to the date of death. OS curves were constructed using Kaplan–Meier method, and differences were analyzed using log-rank test with SPSS v.25 statistical software (IBM).

### SRB assay

For sulforhodamine B (SRB) assay, cells were seeded in 96-well flat bottom plates at a density of 3000–4000 cells/well. After 72 h of drug exposure, plates were fixed with 50% TCA, and incubated with 0.4% SRB at room temperature avoiding light. Plates were washed with 1% acetic acid to remove unbound SRB and air dried. 10 mM Tris was used to extract protein-bound dye and optical density was measured with a BioTek Synergy HT plate reader (SN 269140, BioTek Instruments Inc.) at 490 and 540 nm. IC_50_ was determined through interpolation in GraphpadPrism (version 9.0, Intuitive Software for Science, USA).

### RT-qPCR and DNA-qPCR

For RT-qPCR, RNA was isolated with an RNEasy Plus Mini kit (QIAGEN, Cat. 74136). 800 ng RNA was used to generate cDNA with the Thermo Scientific RevertAid H Minus First Strand cDNA Synthesis Kit (Thermo Fisher Scientific, Waltham, MA, USA). For DNA-qPCR, genomic DNA was isolated with the GenElute™Mammalian Genomic DNA Miniprep kit, following manufacturer instructions (Cat. GIN350, Sigma-Aldrich, St. Luis, MO, USA). RNAse A solution (provided with the kit, 1:10 dilution) was used to obtain RNA-free DNA. 7.5 ng of genomic DNA was used as template. qPCR was performed in triplicate using the PowerUp™ SYBR™ Green Master Mix (Thermo Fisher Scientific, Waltham, MA, USA) in a QuantStudioTM 6 Flex Real-Time PCR system (Applied Biosystems®, ThermoFisher Scientific, Waltham, MA, USA). Primers were from Sigma-Aldrich (St. Louis, MO, USA) and were directed against exon-exon boundaries for RT-qPCR or directed against intron–exon boundaries or introns for DNA-qPCR (Supplemental Table S1). Relative mRNA expression and relative DNA amount was calculated using the 2^−(ΔΔCt)^ method with *ACTB* and *GAPDH* as reference genes.

### Western blot

Cells were lysed with RIPA buffer supplemented with 1% protease/phosphatase inhibitor cocktail (PIC, Sigma-Aldrich, St. Luis, MO, USA), 40 μg lysates were separated by SDS–polyacrylamide gel electrophoresis and transferred to PVDF membranes. Membranes were incubated overnight at 4 °C with rabbit-anti-human ABCB1 (E1Y7B; mAb #13,342; Cell signaling Technology; dilution 1:1000) and mouse-anti-human β-actin (sc-47778; Santa Cruz Biotechnology, Santa Cruz, CA, USA; dilution 1:1000) antibodies, followed by incubation with HRP-conjugated anti-rabbit (#7074; Cell Signaling Technology; dilution 1:2000) and Alexa Fluor® 647-conjugated anti-mouse (115–605-146; Jackson ImmunoResearch, Bio-Connect, Huissen, The Netherlands; dilution 1:1000) secondary antibodies for 1 h at room temperature. Signals were detected using enhanced chemiluminescence (ECL) and fluorescence readouts.

### Hoechst exclusion assay

Cells were seeded at a density of 7000 cells/well in 96-well flat bottom black imaging plates (#655090; Greiner Bio-One™). Cells were allowed to attach for 8 h and then treated with 1 μM of the selected KI, 10 μM verapamil, or DMSO as negative control. Each condition was tested in triplicate wells. 24 h after seeding, 1 μg/mL Hoechst33342 was added followed by an additional 2-h incubation. Subsequently, all wells were aspirated and received fresh medium including the respective inhibitors, without Hoechst33342 and plates were placed in a Nikon Eclipse Ti confocal microscope equipped with an automated stage, temperature and CO_2_-controlled incubator for live imaging and a Plan Apo ×20/0.75 NA objective (Nikon Instruments Inc., Melville, NY, USA). Total intensity of nuclear Hoechst33342 was calculated with CellProfiler [28] after watershed segmentation in FIJI-ImageJ [29]. The intensity values of three images from three replicate wells were averaged for each condition. Values of experimental groups were normalized to those of the DMSO control group.

### siRNA mediated ABCB1 and Sorcin knockdown

Cells were reverse transfected with 50 nM SMARTpool siGENOME siRNAs (Dharmacon) using INTERFERin transfection reagent (Polyplus; 409–50). A mixture of siRNAs targeting all kinases in the human genome, diluted to a total concentration of 50 nM with a concentration for each individual siRNA ~ 0.05 nM was used as control (siKINASEpool). Medium was refreshed after 24 h. For SRB assays, 3000 cells/well were seeded in triplicate wells in 96-well flat bottom plates. For RT-qPCR, 120,000 cells/well were seeded in duplicate wells in 24-well plates. At 48 h and 72 h post-transfection, cells in 24-well plates were processed for RT-qPCR and cells in 96-well plates were incubated for an additional 72 h in presence of DMSO or paclitaxel and subsequently processed for SRB assay.

### Extrachromosomal DNA analysis

Cells in the exponential growth phase (70% confluent) were treated with colcemid (KaryoMax, #15212012, Gibco™) for 1-2 h at a final concentration of 0.1 μg/ml. Cells were then detached by trypsinization, collected, and treated with a hypotonic solution (75 mM KCl) for 15 min. Next, cells were fixed in Carnoy’s fixative (3:1 Methanol:Glacial acetic acid), washed three times and resuspended in 200 μL of Carnoy’s solution. Finally, metaphase chromosomes were prepared by dropping the cell suspension onto glass slides and mounted with ProLong™ Diamond Antifade mountant containing DAPI (Invitrogen, P36966, Waltham, MA, USA). Chromosomes and extrachromosomal DNA (ecDNA) were visualized with a Nikon Eclipse Ti2 confocal microscope, with a 60X objective and a 2 × digital magnification.

### Kinase inhibitor screening

The L1200 library from Selleckchem® (Munich, Germany) was used, containing 760 KIs that were dissolved in DMSO or water at a concentration of 10 mM or 1 mM. 3000 cells/well were seeded in 96-well flat bottom plates. After 24 h cells were treated with DMSO only (0.1%), 1 μM KI and DMSO, or 1 μM KI in combination with 0.1 μM paclitaxel. 10 μM verapamil was used as a positive control. After 72 h, cells were fixed and analyzed using an SRB-assay. The KI library was screened in single technical replicates, and the experiment was repeated in two biological replicates. Rescreening of selected KIs with SRB assay was performed in duplicate technical replicates and the experiment was repeated in three biological replicates.

### Bottom-up proteomics sample preparation

For bottom-up proteomics, peptides were prepared by lysis of cells with a 5% SDS solution and Roche cOmplete™ Mini EDTA-free Protease Inhibitor Cocktail (Merck Darmstadt, Germany), followed by sonication (10 min, every 30 s) (Bioruptor Pico Diagenode, Belgium). Next, protein lysates were quantified using a modified Pierce Micro BCA assay (Termo Fisher Scientifc Rockford, IL), and 5 μg of proteins were used for peptide digestion. Prior to digestion, proteins were reduced with 20 mM dithiothreitol (Merck Darmstadt, Germany) at 45 °C for 30 min, alkylated with 40 mM iodoacetamide (Merck Darmstadt, Germany) for 30 min at room temperature in the dark and acidified with 2.5% phosphoric acid. Finally, proteins were diluted with 90% methanol/100 mM triethylammonium bicarbonate (TEAB) (Merck Darmstadt, Germany) for efficient trapping in Micro S-Trap columns (ProtiFI, Farmingdale, NY, USA). Digestion was performed in the S-Trap overnight at 37 °C using Trypsin/Lys-C Mix Mass Spec Grade (Promega, Walldorf, Germany), followed by elution in 50 mM TEAB, 0.2% formic acid (FA) and 50% acetonitrile (ACN) (Merck Darmstadt, Germany). Eluted peptides were dry-evaporated and resuspended in 10% FA solution for subsequent tandem mass spectrometry analysis (LC–MS/MS), as described in Supplementary Methods. The MS proteomics data have been deposited to the ProteomeXchange Consortium via PRIDE (accession number PXD040930).

### RNA-seq

Total RNA was extracted from cells using the MiRVAna kit (Ambion, Thermo Fisher Scientific). Library preparation was performed using the Illumina TruSeq Stranded total RNA Library Prep gold Kit (20020598, Illumina Inc., San Diego, USA) and Agencount AMPure XP beads (Beckman Coulter, Brea, USA). Library concentration was determined using a Qubit dsDNA BR kit (Thermo Scientific), and the size distribution was examined with an Agilent Bioanalyzer. Libraries were paired-end sequenced (2 × 75 bp) on a NextSeq500 (Illumina). BclToFastq was used for the preprocessing of the raw data (trimming and filtering), then FASTQ files were checked for read quality and adapters were removed with Trimmomatic. The resulting reads were then mapped to the human reference genome (GRCh38) with STAR mapping tool (version 2.5.3a) and gene counts extracted with HTSeq. Raw RNA-sequencing data have been deposited on GEO database under accession number GSE228106.

### Differential expression analysis

Differential expression analysis was performed with R package Deseq2 (version 1.22.2) for RNA-seq data and R package Limma (version 3.38.3) for proteomics data. In all datasets, black and white cases were allowed retaining the 0 for both parental and resistant cells. For RNA-seq data, only genes having a total sample count > 10 were retained. Volcano plots were generated using R package *EnhancedVolcano*, principal component analysis and sample correlation analyses were performed with *plotPCA* function of DeSeq2 R package and *pheatmap* R package (version 1.0.12). Finally, the R-package *ggvenn* was used to count the genes significantly upregulated in common among the cell lines and between RNA-seq and proteomics datasets.

### Statistical analysis

Experiments were performed at least 3 times and data are expressed as mean ± SD of 3 experiments performed in triplicate, unless otherwise specified. To compare between two groups a two-tailed unpaired Student’s t-test was used. For multiple groups comparisons an ordinary one-way ANOVA multiple comparison test with Dunnet’s post-hoc test was used, unless otherwise specified in figure legends. Statistical significance was set at *p* < 0.05 and is indicated by *, *p* < 0.05; **, *p* < 0.01; ***, *p* < 0.001; ****, *p* < 0.0001.

## Results

### Establishment of PDAC resistant cell lines

**Fig. 1:**
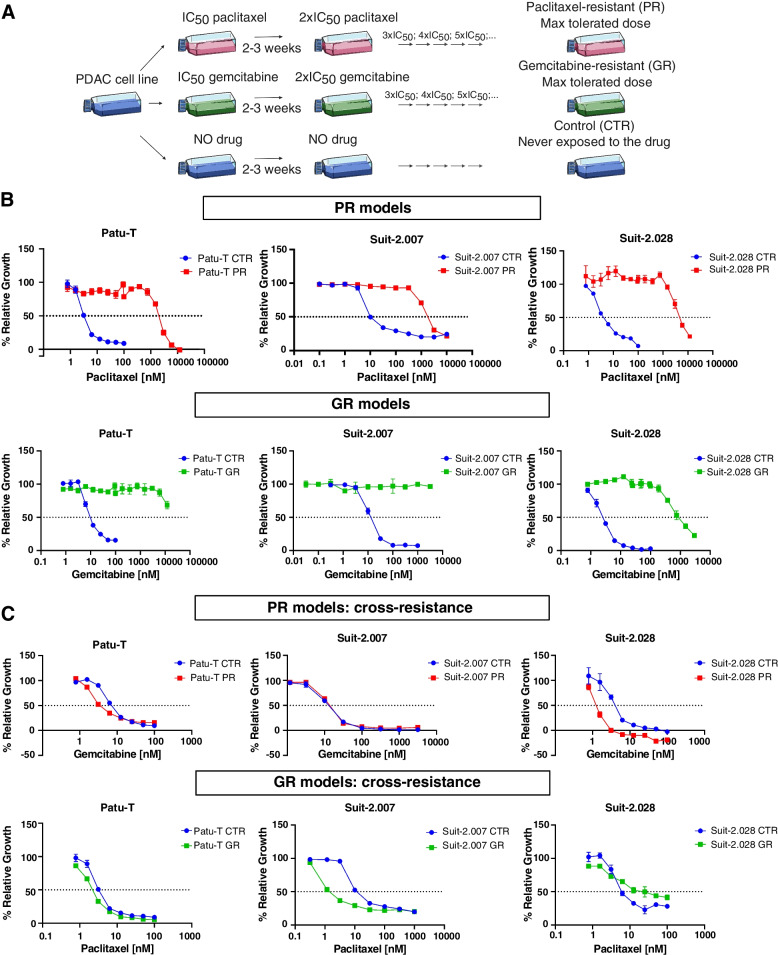
Establishment of paclitaxel and gemcitabine resistant PDAC models. **A **Graphical representation of the methodology used for generation of PR and GR models. **B** Growth curves of PR (upper panel) and GR (lower panel) cells, together with the parental cells, exposed to increasing concentrations of gemcitabine or paclitaxel. **C **Sensitivity of PR cells to gemcitabine and GR cells to paclitaxel. **B**-**C **Mean and SD of triplicates are shown. IC_50_ values were calculated as mean of 3 independent experiments, each performed in triplicate

To study resistance to paclitaxel in PDAC, resistant cell lines were established by adaptation to a stepwise increase in the exposure dose over the course of 6–12 months (Fig. 1A). For this purpose, Patu-T, Suit-2.007, and Suit-2.028 cell models were used, and gemcitabine was used as an alternative chemotherapy in parallel to paclitaxel. The IC_50_ values, as extrapolated from the dose–response curves for paclitaxel or gemcitabine, ranged from 2 to 16 nM for all parental cell lines (Fig. 1B and Supplemental Table S2). IC_50_ values for the resistant derivatives GR and PR were in the μM range (1.6–3.0 μM for PR; 0.7–12 μM for GR), with resistance factors > 100-fold that remained stable for at least 2 months of culturing in absence of the drug (Fig. 1B and Supplemental Table S2). We did not observe cross-resistance: GR cells showed similar or even greater sensitivity to paclitaxel as compared to parental cells and PR cells showed similar or even greater sensitivity to gemcitabine as compared to parental cells (Fig. 1C and Supplemental Table S2). All together these results indicated that resistant models were stable and did not show cross-resistance, therefore providing a valid model to investigate drug-specific chemoresistance mechanisms.

### ABCB1 expression is induced in PR but not GR models

**Fig. 2:**
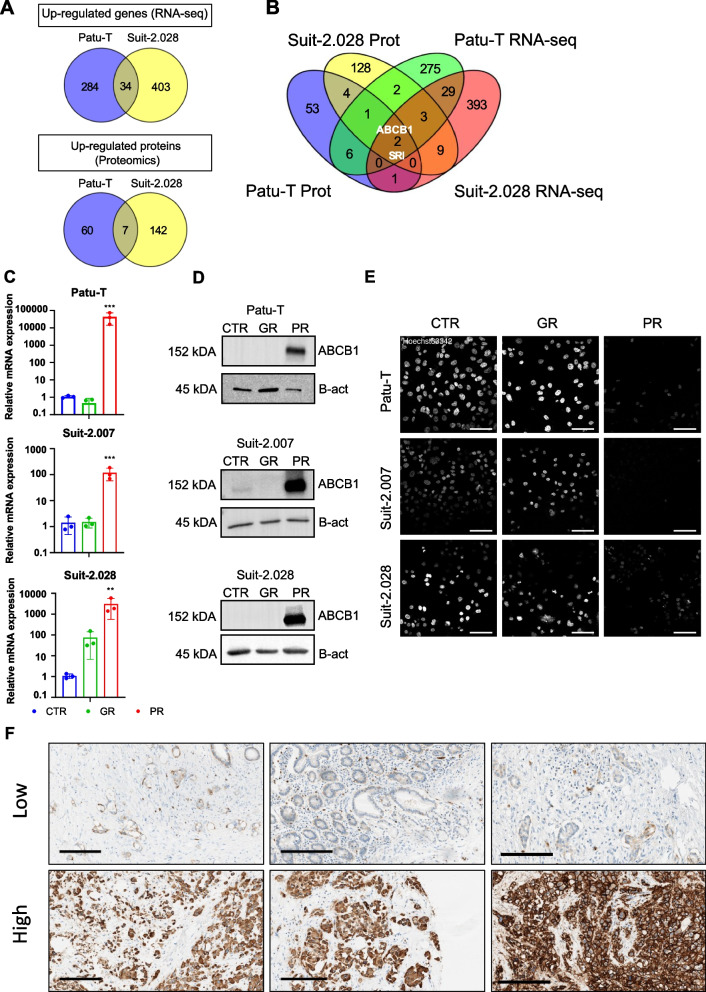
ABCB1 overexpression in PDAC PR cell lines. **A **Venn diagrams showing the up-regulated mRNAs (upper panel) or proteins (lower panel) in Patu-T PR and Suit-2.028 PR compared to the respective CTR. A list of the genes in common for each dataset can be found at Supplemental Table S3. **B **Venn diagram showing targets upregulated in both RNA-seq (Patu-T PR in green and Suit-2.028 in red) and proteomics analysis (Patu-T PR in blue and Suit-2.028 in yellow) in Patu-T PR and Suit-2.028 PR compared to the respective CTR. **C **Relative gene expression of *ABCB1* in CTR (blue), GR (green) and PR (red), measured by RT-qPCR. **D **Western blot analysis of ABCB1 expression and β-actin (B-act) as loading control in the indicated CTR, GR, and PR cell models. Uncropped Western blot membranes can be found in Figure S4. **E **Representative confocal images of PDAC cell lines stained with 1 μg/mL of Hoechst33342 for 2 h at 37 °C in growth medium. *Scale bar:* 100 μm. **F **ABCB1 expression levels assessed by immunohistochemistry (IHC) in surgical specimens from PDAC patients who were subsequently treated with gemcitabine/nab-paclitaxel as first-line therapy. Three specimens with low and three specimens with high expression levels are shown. *Scale bar*: 200 μm

To investigate a common molecular mechanism for paclitaxel resistance in PR cells, Patu-T and Suit-2.028 parental (CTR) and PR cells were subjected to RNA-seq and proteomics analysis. Both RNA-seq and proteomics were performed in triplicate and correlation plot and principal component analyses showed a good separation among CTR and resistant cells (Fig. S1A, B). For differential expression of RNA and proteins, cutoff criteria were set at log2FC < -2 or > 2 and *p*-val < 0.05. RNA-seq analysis identified 720 upregulated genes in PR cells (284 unique for Patu-T; 403 unique for Suit-2.028; 34 in common) (Fig. 2A and Fig. S2). Proteomics analysis identified a total of 5309 (Patu-T) and 5231 (Suit-2.028) unique proteins and 209 proteins were upregulated in PR cells (60 unique for Patu-T; 142 unique for Suit-2.028; 7 in common). Intersection of RNA-seq and proteomics data identified ABCB1 and SRI as the only genes whose expression was upregulated both at the transcript and protein level in both PR models (Fig. 2B and Supplemental Table S3). Other ABC transporters were not upregulated in both cell lines and in both RNA-seq and proteomics data sets (Fig. S3). We validated upregulation of ABCB1 by RT-qPCR and Western Blot. ABCB1 was strongly upregulated at the mRNA and protein level in all PR cell lines, as compared to GR and parental cell lines (Fig. 2C, D; Fig. S4). Even though Suit-2.028 GR cells showed some increase in ABCB1 mRNA expression, ABCB1 protein levels were not affected. These findings indicated that induction of ABCB1 is a common event in PR PDAC models but not in GR models.

### ABCB1 represents a target for sensitization to paclitaxel in PDAC

**Fig. 3:**
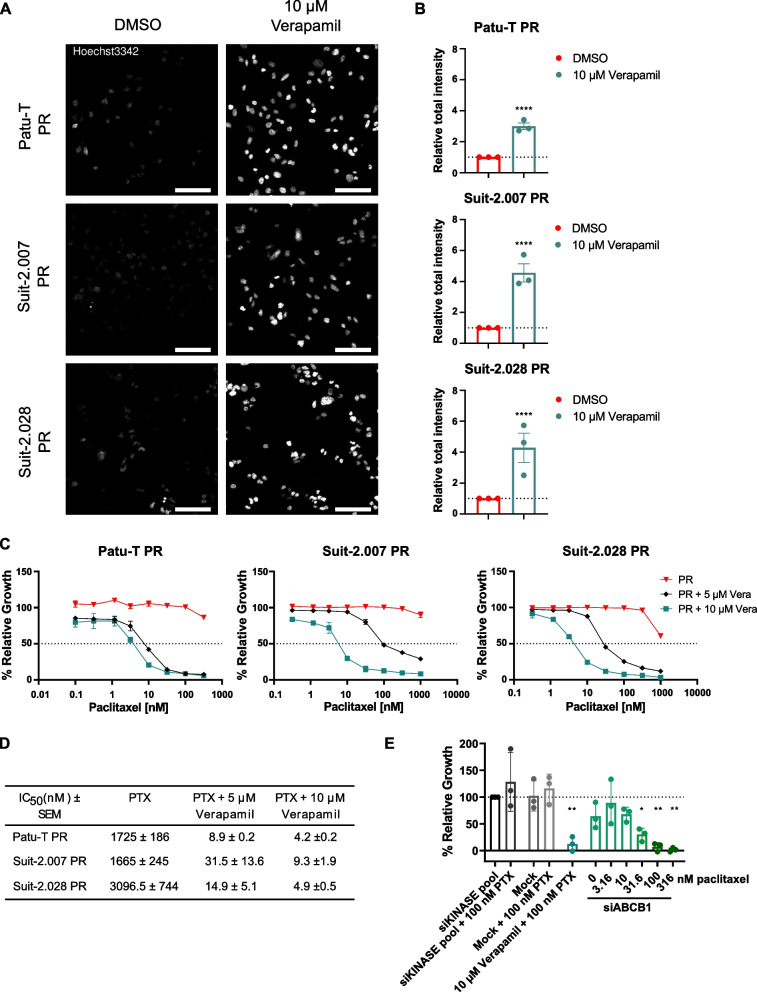
ABCB1 inhibition restores PR cell lines paclitaxel sensitivity. **A **Representative confocal images of PDAC PR cell lines treated O/N with either 10 μM verapamil or DMSO as a control and stained with 1 μg/mL of Hoechst33342 for 2 h at 37 °C in growth medium. *Scale bar:* 100 μm. **B **Quantification of Hoechst signal total intensity with CellProfiler upon DMSO (red) or 10 μM verapamil (green) treatment. **C **Representative growth curves of the 3 PR resistant cell models exposed for 72 h to paclitaxel concentration ranges, combined with 0.1% DMSO (red triangles), 5 μM verapamil (black diamonds) or 10 μM verapamil (green squares). Mean and SD of triplicates is shown. **D **Concentrations of paclitaxel causing 50% reduction in cell growth determined in absence or presence of 5 μM or 10 μM verapamil. Mean ± SEM for 3 independent experiments is displayed. As 50% growth inhibition was not fully reached in PR cells exposed to only paclitaxel, values from Supplemental Table S2 are displayed. **E **Relative proliferation (compared to siKINASEpool control) of Patu-T PR cells 72 h post-treatment with the indicated siRNA SMARTpools (50 nM) and paclitaxel concentrations, analyzed by SRB assay. Verapamil is used as positive control for ABCB1 inhibition

The functional consequence of increased ABCB1 expression was determined using a Hoechst-efflux assay. Similarly to paclitaxel, the live nuclear stain Hoechst33342 is a substrate of multiple ABC-transporters, including ABCB1 [30]. Hoechst33342 readily stained nuclei in CTR and GR cells but was effectively excluded in PR cells after 2 h incubation (Fig. 2E). To confirm the clinical relevance of ABCB1, its expression and potential correlation with survival was evaluated in surgical specimens from PDAC patients who then received at least one cycle of gemcitabine + nab-paclitaxel as first-line therapy. ABCB1 was expressed at different levels in these patients confirming its potential role as a personalized target for therapeutic intervention in PDAC patients (Fig. 2F). There was a trend towards a correlation with poor survival although in this small cohort this was not significant (*p* = 0.0694, Fig. S5A, B). To establish the role of ABCB1 in PDAC paclitaxel-resistance, ABCB1 activity was inhibited with the ABCB1 inhibitor, verapamil [31]. Treatment with verapamil alone did not affect PR cell proliferation (Fig. S6A), but led to a marked increase in Hoechst nuclear staining in PR cells (Fig. 3A, B). In agreement, a dose-dependent increase in the sensitivity to paclitaxel was observed causing a ~ 100–1000-fold decrease in the paclitaxel IC_50_ when combined with 10 μM verapamil (Fig. 3C, D). Moreover, verapamil did not affect gemcitabine sensitivity in Patu-T GR or CTR (Fig. S6B). As verapamil may have off-target effects in addition to ABCB1 inhibition, the role of ABCB1 in PDAC paclitaxel resistance was further confirmed using gene silencing. Indeed, siRNA-mediated ABCB1 depletion strongly sensitized PR cells to paclitaxel as compared to controls (Fig. 3E). Together, these data confirmed the specific role of ABCB1 induction in paclitaxel resistance as a common mechanism underlying paclitaxel-resistance in all three PDAC models.

### ABCB1 gene locus is amplified and gene expression is upregulated in PR cells

**Fig. 4:**
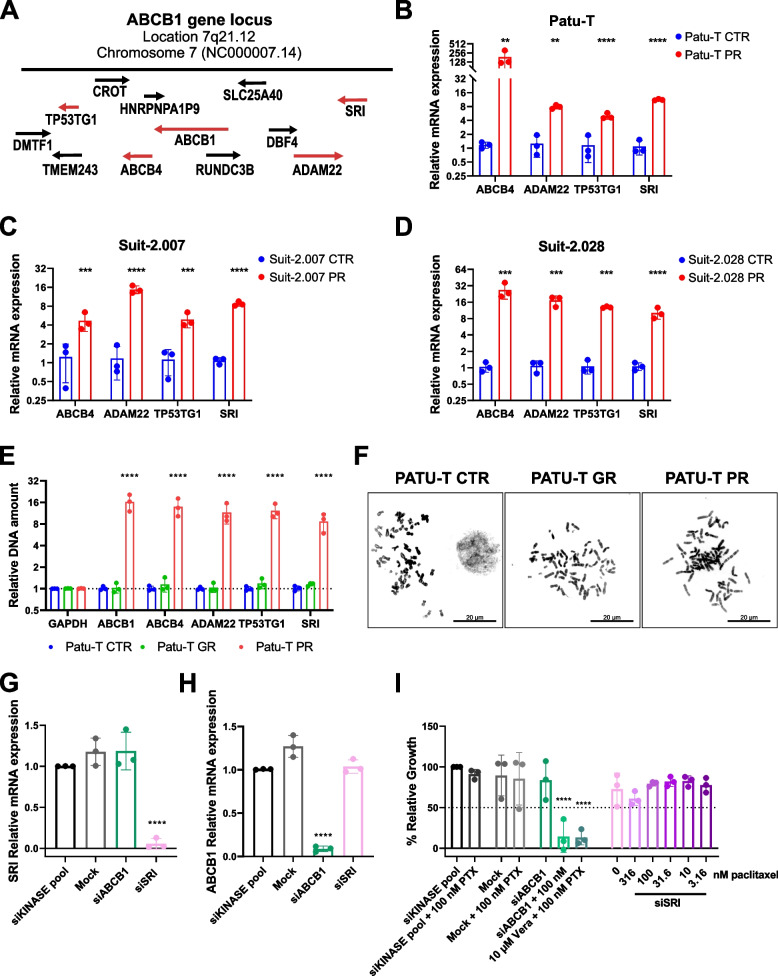
Expression of genes in ABCB1 locus is upregulated in PR cells. **A **Graphic visualization of ABCB1 amplicon on the locus 7q21.12. **B**-**D **Gene expression of *ABCB4, ADAM22, TP53TG1* and *SRI* measured by RT-qPCR in PR (red) relative to CTR cells (blue) for the 3 indicated cell models. Brown-Forsythe and Welch ANOVA with Dunnet’s T3 post hoc test was used **E **Relative DNA amount of *GAPDH*, *ABCB1, ABCB4, ADAM22, TP53TG1* and *SRI* in Patu-T CTR (blue), GR (green) and PR (red) cells, measured by DNA-qPCR and calculated as fold change (2^−ΔΔCt^ compared to the parental). **F **Representative images of Patu-T CTR, GR and PR cell metaphases stained with DAPI. Note absence of ecDNA. *Scale bar:* 20 μm. **G**-**H **Gene expression of *SRI* (**G**) and *ABCB1* (**H**) in Patu-T PR cells transfected with the indicated siRNA SMARTpools (50 nM) measured by RT-qPCR relative to siKINASEpool samples. **I **Cell growth of Patu-T PR cells 72 h post-treatment with the indicated siRNA SMARTpools and paclitaxel concentrations, relative to siKINASEpool control samples. Verapamil (Vera) is used as positive control for ABCB1 inhibition

ABCB1 overexpression can be caused by amplification of the gene locus 7q21.12 in neuroblastoma, lung, and ovarian cancers [32]. To elucidate the mechanism of upregulation in the three PR PDAC models, mRNA expression of the *ABCB4*, *ADAM22*, *TP53TG1*, and *SRI* genes that reside in the ABCB1 locus (Fig. 4A), was measured by RT-qPCR. Expression of each of these genes was increased in PR cells as compared to the parental cells for each of the three PDAC models (Fig. 4B-D), suggesting amplification or de-repression of the gene locus. We explored locus amplification by DNA-qPCR in the Patu-T model. Increased signals for all four genes were detected in PR but not in GR cells as compared to CTR cells, confirming locus amplification (Fig. 4E). We next investigated the presence of ecDNA that has been associated with increased copies of oncogenes and chemo-resistance in many types of cancer [33, 34]. Similar to parental or GR cells, no ecDNA was present in PR cell metaphase spreads (Fig. 4F). This demonstrated that ABCB1 overexpression in PR PDAC cells was caused by ABCB1 locus amplification which does not involve ecDNA.

### Sorcin depletion does not affect proliferation of PR cells treated with paclitaxel

*SRI*, which was the only gene up-regulated at the mRNA and protein level in all three PR PDAC models alongside ABCB1 (Figs. 2B, 4B-D), encodes the calcium-binding protein Sorcin that is associated with cancer progression [35] and can activate expression of ABCB1 [36]. We therefore asked if depletion of SRI could reduce ABCB1 levels and restore paclitaxel sensitivity in PR PDAC cells. However, siRNA-mediated silencing of SRI did not lead to reduced ABCB1 expression (Fig. 4G, H) and, in agreement, did not affect paclitaxel resistance of Patu-T PR cells (Fig. 4I). This indicates that a previously described mechanism of ABCB1 regulation by SRI did not underlie ABCB1-mediated paclitaxel resistance in PDAC cells.

### Compound screen identifies KIs targeting ABCB1-mediated paclitaxel resistance

**Fig. 5:**
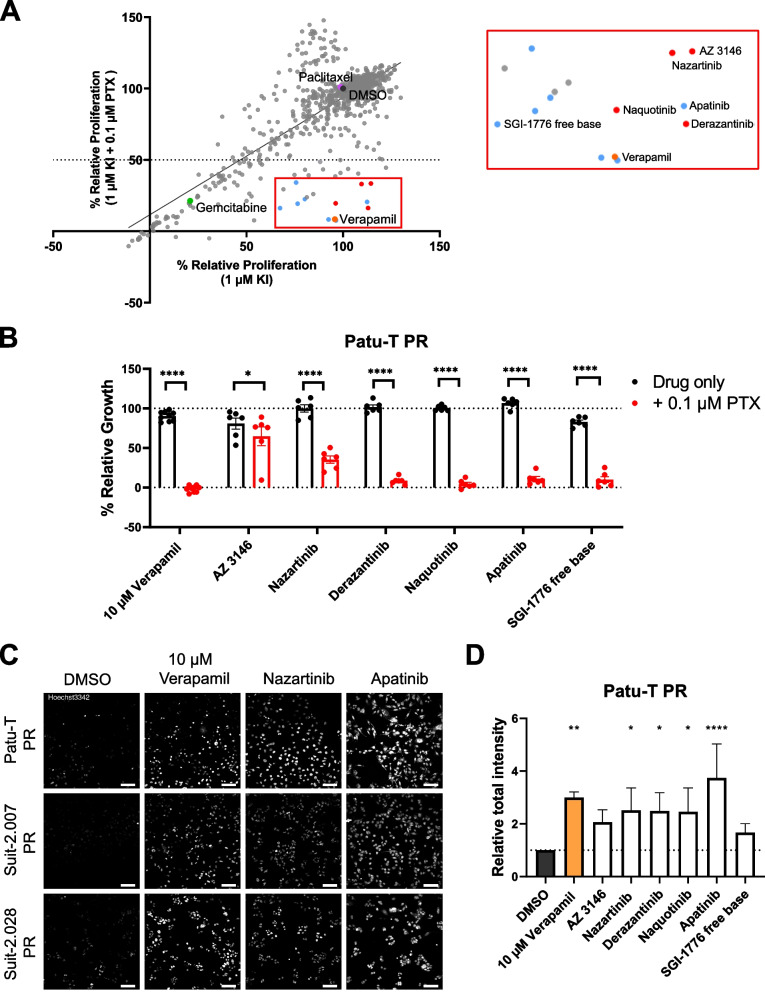
KI library screen to identify synthetic lethalities with paclitaxel in PR PDAC cells. **A **Scatter plot showing relative proliferation of Patu-T PR cells treated with 760 KIs (1 μM) in absence or presence of 0.1 μM paclitaxel, as assessed by SRB. Dots represent the mean of two independent experiments. Labeled dots indicate 0.1% DMSO control (dark grey), 0.1 μM paclitaxel only (purple), 0.1 μM gemcitabine (green), and 10 μM verapamil (orange). Red box, enlarged on the right, indicates compounds synergizing with paclitaxel, and blue dots indicate compounds already known to interact with ABCB1. **B **Confirmation of screen hits. KIs were tested at 1 μM concentration in combination with DMSO (black) or 0.1 μM paclitaxel (red) and proliferation was assessed after 72 h of treatment. Dotted line represents DMSO control (100%). KIs were tested in technical duplicates and controls in triplicates. Mean and SD from 3 independent experiments is displayed (dots indicate individual data points). Ordinary one-way ANOVA was performed, followed by Šídák’s multiple comparisons test. **C **Representative confocal images of PDAC PR cell lines treated O/N with either 1 μM KIs, 10 μM verapamil or DMSO as a control and stained with 1 μg/mL of Hoechst33342 for 2 h at 37 °C in growth medium. *Scale bar:* 100 μm. **D **Quantification of Hoechst signal total intensity with CellProfiler upon the different treatments. Dotted line represents Relative total intensity = 1

Clinical trials with ABCB1 inhibitors on cancer patients have not been successful due to low efficacy or adverse effects [21, 27]. As sorcin targeting proved unsuccessful, we took an unbiased approach to identify alternative pharmacological combinations to restore paclitaxel sensitivity in PR PDAC cells. We screened a library of 760 KIs in Patu-T PR cells. The KI library was screened at a fixed dose of 1 μM in combination with DMSO or paclitaxel at a fixed dose of 0.1 μM. A subset of KIs reduced proliferation of Patu-T PR cells below 50% exclusively when co-administered with paclitaxel (Fig. 5A, Supplemental Table S4). This subset did not show an enrichment for interaction with specific signaling pathways but several of these KIs had been previously shown to interact with ABCB1. In particular, tyrosine kinase inhibitors, including apatinib and SGI-1776 free base, have been described as ABCB1 inhibitors [21, 37–39]. We further continued with apatinib and SGI-1776 free base as positive controls and a series of potent KIs detected in the screen for which an interaction with ABCB1 had not been previously shown (Supplemental Table S4). Sensitization to paclitaxel for PR PDAC cells was validated for each of these selected KIs in the Patu-T, Suit-2.028 and Suit-2.007 models (Fig. 5B and Fig. S7A). In agreement with ABCB1 inhibition by these KIs, each effectively suppressed Hoechst exclusion in the PR cells to a similar extent as that achieved by verapamil (Fig. 5C, D and Fig. S7B). To discriminate between inhibition of ABCB1 efflux function versus inhibition of expression of ABCB1, we measured ABCB1 mRNA expression in the PR models after 48 h of treatment with 1 μM of selected KIs. The effect of the KIs varied among cell lines and among KIs, but none of them reduced ABCB1 expression to a level comparable to that in CTR cells (Fig. S8). Moreover, changes in expression induced by KIs did not match their effect on Hoechst exclusion or cell proliferation in the presence of paclitaxel, indicating that these KIs primarily act as inhibitors of ABCB1 function. Altogether, these findings identify novel KIs that may be used to target ABCB1-mediated paclitaxel resistance.

## Discussion

Chemoresistance is a major hurdle in the treatment of PDAC patients and understanding how to revert resistance to available treatments is of crucial importance. Gemcitabine resistance has been extensively investigated in PDAC, while little research has been performed on paclitaxel resistance [12, 13]. We find that ABCB1 overexpression is a shared response to continued exposure to paclitaxel in three independent PDAC models and is not associated with gemcitabine resistance in those models. ABCB1 is involved in multidrug resistance of many solid cancers [27, 40–42]. Taxols, in particular paclitaxel, are among the substrates of this transporter. However, the role of ABCB1 in resistance to paclitaxel has not been addressed in pancreatic cancer.

Interestingly, previous studies using pancreatic and other cancer cell lines have shown that ABCB1 is involved in gemcitabine resistance [13, 22–26]. Our data do not support such a role: gemcitabine exposure did not induce ABCB1 expression (besides some increase at the mRNA level in some instances which was not mirrored by enhanced protein levels). Moreover, the induction of ABCB1 in paclitaxel resistant PDAC cells did not lead to cross-resistance to gemcitabine and inhibition of ABCB1 through verapamil did not alter GR cells sensitivity to gemcitabine. The aforementioned studies largely focused on HNF1A or PLK1 mediated gemcitabine resistance mechanisms that involved ABCB1 [22, 23, 25] while Chen et al. did observe increased ABCB1 expression in SW1990 cells treated with gemcitabine [24]. Notably, a different study in fact reported increased sensitivity to gemcitabine in a panel of cancer cell lines overexpressing ABCB1 [43], and we find a similar trend in some of our models. Taken together, there is no direct evidence involving ABCB1 in gemcitabine resistance and our study indeed argues against such a mechanism in PDAC cells.

Previous studies showed that ABCB1 overexpression can be caused by gene locus amplification [44]. Indeed, the expression of 4 genes belonging to locus 7q21.12 (*ABCB4*, *ADAM22*, *TP53TG1* and *SRI*) is also increased in PR cells. We discriminate between de-repression and amplification by DNA-qPCR, further confirming locus amplification as the underlying mechanism. Gene amplification and chemoresistance have been linked to the presence of ecDNA [33]. The continuous exposure to a drug like paclitaxel affecting the cell cycle could lead to genomic instability and therefore to the generation of ecDNA fragments [45] but we did not find evidence for this. The fact that DNA-qPCR fold-change values were similar for the tested genes in the 7q21.12 locus, while RT-qPCR fold-change values for the same genes differed considerably, suggests that additional mechanisms, on top of locus amplification, may regulate paclitaxel-induced ABCB1 overexpression.

One such mechanism we considered, involves SRI/Sorcin. *SRI* is located on the same gene locus as *ABCB1* and is often co-amplified in multidrug-resistant cancers [32, 35]. In our experiments, SRI was the only candidate specifically induced by paclitaxel along with ABCB1 in the transcriptomics and proteomics datasets for Patu-T PR and Suit-2.028 PR. SRI encodes Sorcin, a calcium-binding protein that has been associated with increased tumor aggressiveness [35, 46] and can induce ABCB1 expression in leukemia [36]. In particular, Sorcin activates Protein Kinase A (PKA)-CREB1 signaling leading to activation of the ABCB1 promoter at cAMP-Response elements (CRE). Our results argue against this mechanism in PDAC paclitaxel resistance: SRI knockdown did not affect ABCB1 expression and failed to sensitize Patu-T PR cells to paclitaxel. It is possible that ABCB1 regulation is different in PDAC cells as compared to leukemic cells or the gradual increase in ABCB1 and SRI caused by paclitaxel differs from engineered SRI overexpression, as used by Yamagishi et colleagues [36]. Moreover, the impact of sorcin on ABCB1 transcription is modulated by the presence of other mechanisms regulating calcium ion homeostasis, which may vary between cell types [47].

Our findings in a small patient cohort show that ABCB1 is expressed in PDAC patients that after resection received at least one cycle of gemcitabine + nab-paclitaxel treatment. We observe a trend towards a correlation of ABCB1 expression with poor survival, although in this small cohort it was not statistically significant. It will be interesting to assess a larger cohort and compare to patients that have received a different therapy regimen such as FOLFIRINOX. Nevertheless, this indicates ABCB1 is expressed and may represent a target for chemosensitization to paclitaxel in PDAC patients. We confirmed its role as a candidate target for attenuating paclitaxel resistance in PDAC using gene silencing and the ABCB1 inhibitor, verapamil. Unfortunately, verapamil or other ABCB1 inhibitors have not performed well in the clinic. Reasons for the failure of ABCB1 inhibitors in clinical trials include lack of efficacy or dose-limiting toxicity [21, 27].

Our KI screen identified novel candidate strategies to sensitize PDAC cells to paclitaxel without affecting growth in the absence of the chemotherapy. We found two compounds (i.e., apatinib and SGI-1776 free base) that have already been reported as ABCB1 inhibitors [38, 39]. Interestingly, several compounds that were identified as ABCB1 inhibitors in different cancers failed to sensitize PDAC cells in our screen (i.e., erlotinib [48], imatinib [49], and nilotinib [50]). Whether this reflects different inhibitory mechanisms or differences in potency is currently unknown, but it underscores the need to test each drug in the appropriate cancer type. We also identified KIs such as nazartinib, naquotinib, and derazantinib, that have not been previously implicated in PDAC paclitaxel resistance or ABCB1 inhibition. Using a Hoechst efflux assay, we confirmed these KIs act by inhibiting ABCB1. For some KIs and in some PR models, inhibition of ABCB1 expression was observed. However, this never reduced it to the nearly absent levels observed in CTR cells. Moreover, this effect did not correlate with the efficacy of the KIs in attenuating Hoechst efflux or cell proliferation in the presence of paclitaxel. This indicates that these KIs mainly act by suppressing ABCB1 efflux activity and it points to candidate strategies for combination therapies for chemosensitization. Interestingly, nazartinib, naquotinib, and derazantinib have already passed phase I clinical trials [NCT02108964 [51], NCT02500927 [52], NCT03230318 [53]], suggesting that their safety profile is acceptable. Apatinib mono treatment showed in vivo tumor growth arrest in PDAC xenograft models [54] and synergized with paclitaxel in gastric cancer murine models [55, 56]. Apatinib also reverted breast cancer multidrug resistance in vivo [38] and it is being tested in phase I and II clinical trials in combination with different chemotherapeutic agents, including paclitaxel [NCT02697838 [57]]. Results from these trials will provide more information on the clinical relevance and feasibility of combining KIs with ABCB1 substrates to improve patient response to therapy.

### Supplementary Information

**Additional file 1:** **Supplementary methods. **LC-MS/MS analysis and label-free quantification. 

**Additional file 2:** **Supplemental Table S1.** Primer sequences used for RT-qPCR and DNA-qPCR.

**Additional file 3:** **Supplemental Table S2.** IC_50_ values of the established resistant cell lines and CTR and the respective Resistance factors. 

**Additional file 4:** **Supplemental Table S3.** List of upregulated RNAs and proteins shared between Patu-T PR and Suit-2.028 PR cells. Common hits in RNA-seq and proteomics data are indicated in bold. 

**Additional file 5:** **Supplemental Table S4.** Selected hits from KI-screen.

**Additional file 6:** **Supplementary Fig. S1. **Correlation plots and Principal component analysis (PCA) of RNA-seq and proteomics data. **A**. Correlation plots show good correlation for biological replicates within the same cell line, and no or poor correlation among different cell lines. **B**. PCA analysis for RNA-seq and proteomics data. Plots show separation of CTR, GR, and PR samples in RNA-seq (top) and proteomics (bottom) data sets. 

**Additional file 7:** **Supplementary Fig. S2. **Volcano plots of differentially expressed genes/proteins for PR vs CTR cells. Grey = not significant (NS); green = only log2FC > 2 or < -2; blue = only *p*-value < 0.05; red = *p*-value < 0.05 and log2FC > 2 or < -2. The latter criteria identified 15198 and 15621 differentially expressed RNAs and 5309 and 5231 differentially expressed proteins in Patu-T and Suit-2.028, respectively. 

**Additional file 8:** **Supplementary Fig. S3. **Upregulation of ABC transporters in PR cells. Venn diagram showing upregulated ABC transporters in PR models.

**Additional file 9:** **Supplementary Fig. S4. **Uncropped Western blot membranes for Patu-T, Suit-2.028 and Suit-2.007 CTR, PR, and GR cells stained for ABCB1 and B-actin**.** One biological replicate of western blot is shown for each cell line. Each sample was collected from untreated cells. 

**Additional file 10:** **Supplementary Fig. S5. **ABCB1 upregulation correlates with poor survival.** A**. Kaplan-Meier curves of the patients that underwent surgery, grouped according ABCB1 expression, showing a trend towards reduced probability of survival in case of high (red) vs. low (blue) expression. **B**. Clinicopathological characteristics and correlation with mean overall survival (OS) of the PDAC patients. 

**Additional file 11:** **Supplementary Fig. S6. **Verapamil mono treatment does not affect PR cell proliferation and verapamil does not sensitize to gemcitabine. **A**. Representative growth curves of 3 PR cell lines (red) exposed to verapamil concentration ranges, relative to DMSO control. Mean and SD of triplicates is shown. The experiment was repeated 3 times. **B**. Representative growth curves showing effect of 5 µM or 10 µM Verapamil on sensitivity to increasing concentrations of gemcitabine for CTR (left) or GR (right) PDAC cells. Mean and SD of triplicates is shown. The experiment was repeated 2 times. 

**Additional file 12:** **Supplementary Fig. S7. **KI screen validation in Suit-2.007 and Suit-2.028 PR cells.** A**. Impact on cell proliferation. Selected KIs from KI library screen were tested at 1 µM in absence (black bars) or presence (red bars) of 0.1 µM paclitaxel (PTX). Proliferation was assessed after 72 hours of treatment. Dotted line represents the DMSO control (100%). Experiments were performed in technical duplicates. Mean and SD of three independent experiments is shown. Ordinary one-way ANOVA was performed, followed by Šídák’s multiple comparisons test. **,*p* < 0.005; ****, *p* < 0.0001. **B**. Impact on Hoechst exclusion. Selected KIs from KI library screen were tested for their ability to prevent Hoechst exclusion in PR cells. Relative Hoechst signal intensity for the indicated treatments versus DMSO is shown. Mean and SD of 3 independent experiments performed in triplicates is shown. Ordinary one-way ANOVA with Dunnet’s post hoc test was used. *, *p* < 0.05; **, *p* < 0.005; ****, *p* < 0.0001. 

**Additional file 13:** **Supplementary Fig. S8. **KI treatment of PR cells does not decrease ABCB1 expression to level of CTR cells. Gene expression of ABCB1 and GAPDH in PR cells after 48h of treatment with 1 µM of the indicated KI, measured by RT-qPCR and calculated as fold change (2^-ΔΔCt^ compared to the DMSO control). Untreated CTR sample was included as negative control for ABCB1 expression (blue). *Bars*, mean of triplicates. 1 experiment was performed. 

## Data Availability

The dataset generated by RNA-seq data for this study is available at Gene Expression Omnibus (GEO) database with accession number GSE228106 and are available at the following URL: https://www.ncbi.nlm.nih.gov/geo/query/acc.cgi?acc=GSE228106. The mass spectrometry proteomics data have been deposited to the ProteomeXchange Consortium via PRIDE repository with the dataset identifier PXD040930 and 10.6019/PXD040930. All experimental datasets and documents generated in this study are available upon reasonable request to the corresponding author.

## Abbreviations

ABC: ATP-binding cassette
ABCB1/MDR1/P-gp: ATP-binding cassette B1
B-Act: Beta-Actin
CRE: CAMP-Response Elements
DMSO: Dimethyl sulfoxide
DNA -qPCR: DNA quantitative PCR
ecDNA: Extra-chromosomal DNA
FOLFIRINOX: Folinic acid-fluorouracil-irinotecan-oxaliplatin
GEM: Gemcitabine
GR: Gemcitabine Resistant
IC_50_: Concentration causing 50% reduction in cell growth
IHC: Immunohistochemistry
KI: Kinase Inhibitor
LC–MS/MS: Liquid chromatography Tandem mass spectrometry
log2FC: Log2-Fold Change
NS: Not significant
O/N: Overnight
OS: Overall Survival
PDAC: Pancreatic ductal Adenocarcinoma
PKA: Protein Kinase A
PR: Paclitaxel Resistance
Prot: Proteomics
PTX: Paclitaxel
*p*-val: *P*-value
RNA-seq: RNA-sequencing
RT-qPCR: Reverse transcription quantitative real-time PCR
SD: Standard deviation
SEM: Standard error of the mean
siRNA: Small interfering RNA
SRB: Sulforhodamine B assay
SRI: Sorcin
Vera: Verapamil
CTR: Parental (control) cells
