# Supplementary material for: ABCB1 overexpression through locus amplification represents an actionable target to combat paclitaxel resistance in pancreatic cancer cells
Source: J Exp Clin Cancer Res. 2024 Jan 2;43:4. doi: 10.1186/s13046-023-02879-8 (PMC10759666; doi:10.1186/s13046-023-02879-8)
Supplement: Supplementary file 1 — Additional file 1: Supplementary methods. LC-MS/MS analysis and label-free quantification. [file 13046_2023_2879_MOESM1_ESM.docx]

**SUPPLEMENTARY METHODS**

### **LC-MS/MS analysis and label-free quantification**

Peptide samples were analyzed by nanoLC- MS/MS using an EASY-nLC 1000 interfaced with an Orbitrap Fusion Tribrid mass spectrometer equipped with an EASY spray source (Thermo Fisher Scientific, Waltham, MA, USA). Peptides were loaded onto a PepMap C18 precolumn (2 cm x 75 µm internal diameter (ID), 3 µm particle size, 100 Å pore size) (Thermo Fisher Scientific, Waltham, MA, USA) and separated on a PepMap C18 column (50 cm x 75 µm ID, 2 µm particle size, 100 Å pore size) (Thermo Fisher Scientific, Waltham, MA, USA) heated at 35 °C and with a constant flow rate of 300 nl/min. Peptide separation was carried out using a segmented gradient of 0.1% FA (mobile phase A) and ACN/0.1% FA (mobile phase B) as follows: from 5% to 22% mobile phase B in 104 min, from 22% to 32% in 15 min and from 32% to 90% in 10 min. MS data were acquired in positive ion mode using a spray voltage of 2.0 kV, sheet gas set to 1 to minimize neutral contamination and an ion transfer tube temperature of 275 °C. The MS1 survey scan was acquired using the orbitrap (OT) analyzer within a mass range of 375 – 1200 m/z, resolving power of 120.000 FWHM (at 200 m/z), RF lens value of 60%, maximum injection time of 50 ms and maximum ion count of 400000. MS2 was performed using the TopSpeed method in which the most intense precursor ions (2 – 7 charge states and a minimum intensity threshold of 5000) were isolated with an isolation window of 1.6 *m/z* and fragmented by higher energy collisional dissociation (HCD) at a normalized collision energy (NCE) of 27%. The total cycle time was 3 s. Fragment ion detection was performed in the dual-pressure ion trap (IT) with the maximum number ions set to 2000 and a maximum injection time of 300 ms. A dynamic exclusion of 60 s was enabled to avoid the selection of the same precursor ion during its chromatographic elution.

Raw files were uploaded into Proteome Discoverer software (v2.1) (Thermo Fisher Scientific, Waltham, MA, USA) and queried against the human UniprotKB/Swiss-Prot TrEMBL database (202160 sequences, September 2021) using the SEQUEST database search algorithm. Peptide identification was performed using a mass tolerance of 10 ppm and 0.6 Da for precursor and fragment ions respectively, trypsin/Lys-C as endoproteases and up to two missed cleavages. Cysteine carbamidomethylation was set as a static modification (+57.021464 Da) while methionine oxidation (+15.994915 Da) and protein N-Terminal acetylation (+42.010565 Da) were both set as variable modifications. Peptide spectrum matches (PSMs) were determined using a 1% false discovery rate (FDR), using the Percolator module. Protein abundances were exported from Proteome Discoverer and normalized over the sum within each cell line dataset. The equality of variances was assessed, and selected proteins were tested for significance using a Student’s two-tailed t-test. The MS proteomics data have been deposited to the ProteomeXchange Consortium via PRIDE (accession number PXD040930).
