# Supplementary material for: ABCB1 overexpression through locus amplification represents an actionable target to combat paclitaxel resistance in pancreatic cancer cells
Source: J Exp Clin Cancer Res. 2024 Jan 2;43:4. doi: 10.1186/s13046-023-02879-8 (PMC10759666; doi:10.1186/s13046-023-02879-8)
Supplement: Supplementary file 2 — Additional file 2: Supplemental Table S1. Primer sequences used for RT-qPCR and DNA-qPCR. [file 13046_2023_2879_MOESM2_ESM.docx]

| RT-qPCR | Forward | Reverse |
| --- | --- | --- |
| ABCB1 | GGCTACATGAGAGCGGAGGAC | TTCCGTTGCACCTCTCTGGTC |
| ABCB4 | GAAAGGCCAGACACTAGCCC | ACCATCGAGAAGCACTGTCC |
| TP53TG1 | GAGCTGTCCTAACTCTGCGG | GAGGGTTGGGTACCTTCGTG |
| ADAM22 | CCGCGAAGCACAATGCAG | CAACATGAGTCAACTGCGGG |
| SRI | TCCGCTGTATGGTTACTTTGC | GTGCCAGACATATCTCTATCCAG |
| ACTB | ATTGCCGACAGGATGCAGAA | GCTGATCCACATCTGCTGGAA |
| GAPDH | TCGGAGTCAACGGATTTGGT | TTCCCGTTCTCAGCCTTGAC |
| DNA qPCR | Forward | Reverse |
| ABCB1 | CAAGGCAATTCACAGACACAGG | CACTTCAGTTACCCATCTCGAA |
| ABCB4 | AGCCCAAGGGTTTAGGTACTG | CTAAAGGCTGAGACCGCCAG |
| TP53TG1 | CTCAGATTTTGGTGGCAACTTTTCA | GGAAGCAGCCAACAGCAAATTA |
| ADAM22 | TGAGGGAACCAAAAGCTCCC | CATGGCCCCTCTACCCTACT |
| SRI | TGTTGGGCTCACATGAAGGT | GGATGGGGGTGCCATTCATT |
| ACTB | CACTCCAAGGCCGCTTTACA | CACTCCAAGGCCGCTTTACA |

**Supplemental Table S1.** Primer sequences used for RT-qPCR and DNA-qPCR.
