# Supplementary material for: ABCB1 overexpression through locus amplification represents an actionable target to combat paclitaxel resistance in pancreatic cancer cells
Source: J Exp Clin Cancer Res. 2024 Jan 2;43:4. doi: 10.1186/s13046-023-02879-8 (PMC10759666; doi:10.1186/s13046-023-02879-8)
Supplement: Supplementary file 3 — Additional file 3: Supplemental Table S2. IC50 values of the established resistant cell lines and CTR and the respective Resistance factors. [file 13046_2023_2879_MOESM3_ESM.docx]

| Cell line |  | IC_50_ Paclitaxel (nM) ± SEM | Paclitaxel resistance factor |  | IC_50_ gemcitabine (nM) ± SEM | Gemcitabine resistance factor |
| --- | --- | --- | --- | --- | --- | --- |
| Patu-T CTR |  | 2.23 ± 0.67 | NA |  | 8.33 ± 1.42 | NA |
| Patu-T PR |  | 1725 ± 186 | **774** |  | 2.10 ± 0.16 | - |
| Patu-T GR |  | 0.92 ± 0.58 | - |  | > 12000 | **1440** |
|  |  |  | |  |  | |
| Suit-2.007 CTR |  | 16.46 ± 6.42 | NA |  | 7.09 ± 2.19 | NA |
| Suit-2.007 PR |  | 1665 ± 245 | **101** |  | 9.78 ± 1.40 | - |
| Suit-2.007 GR |  | 0.53 ± 0.50 | - |  | > 3000 | **423** |
|  |  |  | |  |  | |
| Suit-2.028 CTR |  | 5.17 ± 0.46 | NA |  | 3.86 ± 0.28 | NA |
| Suit-2.028 PR |  | 3096.5 ± 744 | **599** |  | 1.65 ± 0.35 | - |
| Suit-2.028 GR |  | 21 ± 5.2 | - |  | 711 ± 116 | **184** |

**Supplemental Table S2.** IC_50_ values of the established resistant cell lines and CTR and the respective Resistance factors.
