# Supplementary material for: ABCB1 overexpression through locus amplification represents an actionable target to combat paclitaxel resistance in pancreatic cancer cells
Source: J Exp Clin Cancer Res. 2024 Jan 2;43:4. doi: 10.1186/s13046-023-02879-8 (PMC10759666; doi:10.1186/s13046-023-02879-8)
Supplement: Supplementary file 4 — Additional file 4: Supplemental Table S3. List of upregulated RNAs and proteins shared between Patu-T PR and Suit-2.028 PR cells. Common hits in RNA-seq and proteomics data are indicated in bold. [file 13046_2023_2879_MOESM4_ESM.docx]

| **Upregulated RNAs in two PR models** | | **Upregulated proteins in two PR models** |
| --- | --- | --- |
| HNRNPA1P9 | DMTF1 | **ABCB1** |
| **ABCB1** | ABCB4 | EEF1A2 |
| AC003991.3 | ADAM22 | FAM83H |
| CROT | MAPK15 | **SRI** |
| CTD-2369P2.8 | TP53TG1_2 | SRXN1 |
| STEAP4 | TP53TG1_1 | TBC1D13 |
| RP11-354M1.2 | FDPSP7 | TMEM120A |
| GRM3 | CTB-167B5.1 |  |
| HOXC13-AS | MDH2 |  |
| AC005522.7 | CACNA2D1 |  |
| AC005076.5 | SEMA3C |  |
| AC034228.4 | POR |  |
| DBF4 | TMEM243 |  |
| KIAA1324L | AC005559.3 |  |
| **SRI** | HLA-DQB1-AS1 |  |
| RP11-66B24.9 | RP11-701H16.4 |  |
| RP11-709A23.2 | AP001610.5 |  |

**Supplemental Table S3.** List of upregulated RNAs and proteins shared between Patu-T PR and Suit-2.028 PR cells. Common hits in RNA-seq and proteomics data are indicated in bold.
