# Supplementary material for: ABCB1 overexpression through locus amplification represents an actionable target to combat paclitaxel resistance in pancreatic cancer cells
Source: J Exp Clin Cancer Res. 2024 Jan 2;43:4. doi: 10.1186/s13046-023-02879-8 (PMC10759666; doi:10.1186/s13046-023-02879-8)
Supplement: Supplementary file 5 — Additional file 5: Supplemental Table S4. Selected hits from KI-screen. [file 13046_2023_2879_MOESM5_ESM.docx]

| Name | Pathway | Target | Literature connection ABCB1 |
| --- | --- | --- | --- |
| AZ 3146 | Cytoskeletal Signaling | Mps1 | no |
| Nazartinib (EGF816. NVS-816) | Angiogenesis | EGFR | no |
| Derazantinib (ARQ-087) | Protein Tyrosine Kinase | FGFR | no |
| Naquotinib (ASP8273) | Angiogenesis | EGFR | no |
| Apatinib | Protein Tyrosine Kinase | VEGFR.c-RET | Yes (Mi Y. 2010) |
| SGI-1776 free base | JAK/STAT | Pim | Yes (Mumenthaler S. 2010) |

**Supplemental Table S4.** Selected hits from KI-screen.
