# Supplementary material for: ABCB1 overexpression through locus amplification represents an actionable target to combat paclitaxel resistance in pancreatic cancer cells
Source: J Exp Clin Cancer Res. 2024 Jan 2;43:4. doi: 10.1186/s13046-023-02879-8 (PMC10759666; doi:10.1186/s13046-023-02879-8)
Supplement: Supplementary file 6 — Additional file 6: Supplementary Fig. S1. Correlation plots and Principal component analysis (PCA) of RNA-seq and proteomics data. A. Correlation plots show good correlation for biological replicates within the same cell line, and no or poor correlation among different cell lines. B. PCA analysis for RNA-seq and proteomics data. Plots show separation of CTR, GR, and PR samples in RNA-seq (top) and proteomics (bottom) data sets. [file 13046_2023_2879_MOESM6_ESM.pdf]

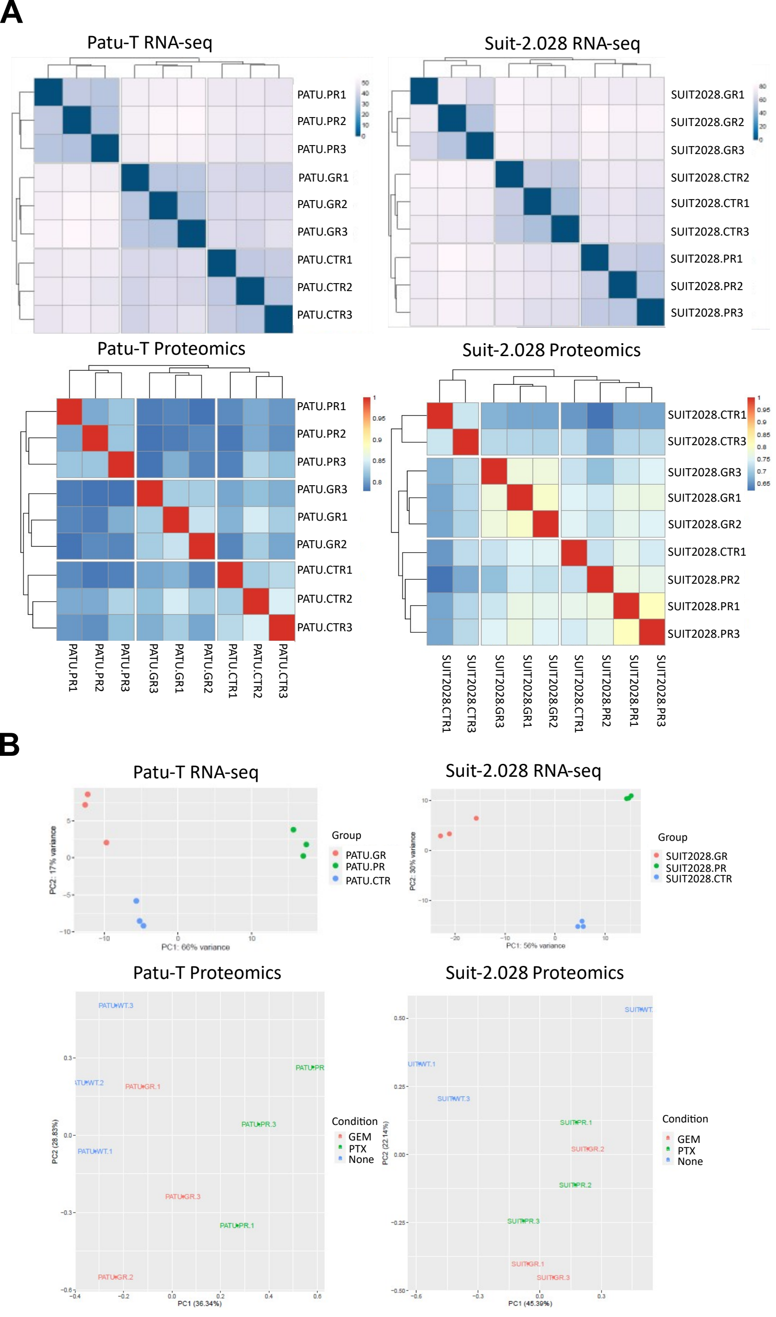
A
Patu-T RNA-seq
PATU.PR1
PATU.PR2
PATU.PR3
PATU.GR1
PATU.GR2
PATU.GR3
PATU.CTR1
PATU.CTR2
PATU.CTR3
Suit-2.028 RNA-seq
SUIT2028.GR1
SUIT2028.GR2
SUIT2028.GR3
SUIT2028.CTR2
SUIT2028.CTR1
SUIT2028.CTR3
SUIT2028.PR1
SUIT2028.PR2
SUIT2028.PR3
Patu-T Proteomics
PATU.PR1
PATU.PR2
PATU.PR3
PATU.GR3
PATU.GR1
PATU.GR2
PATU.CTR1
PATU.CTR2
PATU.CTR3
Suit-2.028 Proteomics
SUIT2028.CTR1
SUIT2028.CTR3
SUIT2028.GR3
SUIT2028.GR1
SUIT2028.GR2
SUIT2028.CTR1
SUIT2028.PR2
SUIT2028.PR1
SUIT2028.PR3
B
Patu-T RNA-seq
Group
PATU.GR
PATU.PR
PATU.CTR
PC1: 66% variance
PC2: 17% variance
Suit-2.028 RNA-seq
Group
SUIT2028.GR
SUIT2028.PR
SUIT2028.CTR
PC1: 56% variance
PC2: 30% variance
Patu-T Proteomics
Condition
GEM
PTX
None
PC1 (36.34%)
PC2 (28.83%)
Suit-2.028 Proteomics
Condition
GEM
PTX
None
PC1 (45.39%)
PC2 (22.14%)

Fig. S1
