# Supplementary material for: ABCB1 overexpression through locus amplification represents an actionable target to combat paclitaxel resistance in pancreatic cancer cells
Source: J Exp Clin Cancer Res. 2024 Jan 2;43:4. doi: 10.1186/s13046-023-02879-8 (PMC10759666; doi:10.1186/s13046-023-02879-8)
Supplement: Supplementary file 7 — Additional file 7: Supplementary Fig. S2. Volcano plots of differentially expressed genes/proteins for PR vs CTR cells. Grey = not significant (NS); green = only log2FC > 2 or < -2; blue = only p-value < 0.05; red = p-value < 0.05 and log2FC > 2 or < -2. The latter criteria identified 15198 and 15621 differentially expressed RNAs and 5309 and 5231 differentially expressed proteins in Patu-T and Suit-2.028, respectively. [file 13046_2023_2879_MOESM7_ESM.pdf]

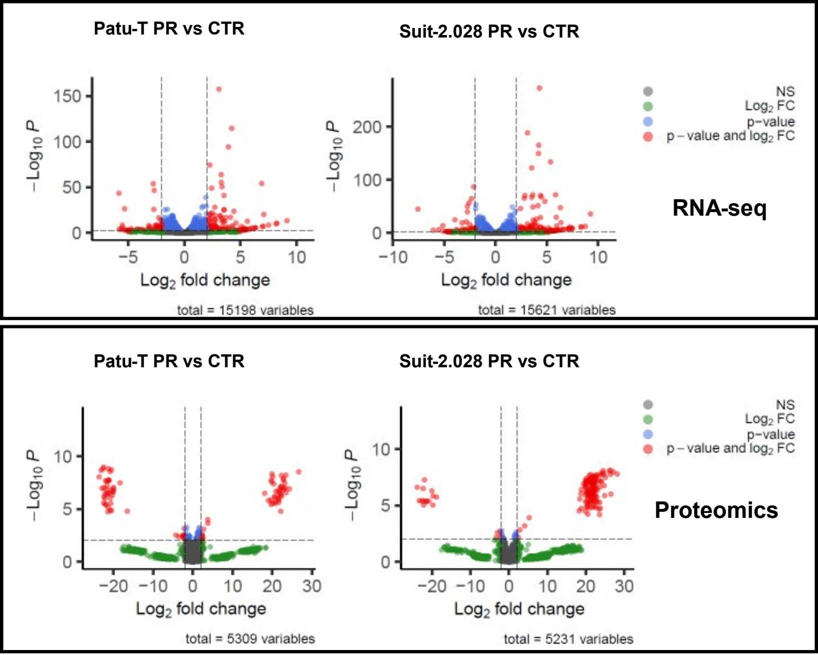
Patu-T PR vs CTR
Suit-2.028 PR vs CTR
NS
Log2 FC
p−value
p − value and log2 FC
RNA-seq
−Log10 P
Log2 fold change
total = 15198 variables
total = 15621 variables
Patu-T PR vs CTR
Suit-2.028 PR vs CTR
Proteomics
total = 5309 variables
total = 5231 variables

Fig. S2
