# Supplementary figures and images for: ABCB1 overexpression through locus amplification represents an actionable target to combat paclitaxel resistance in pancreatic cancer cells
Source: J Exp Clin Cancer Res. 2024 Jan 2;43:4. doi: 10.1186/s13046-023-02879-8 (PMC10759666; doi:10.1186/s13046-023-02879-8)

## Suit-2.028 RNA-seq

## Patu-T Prot

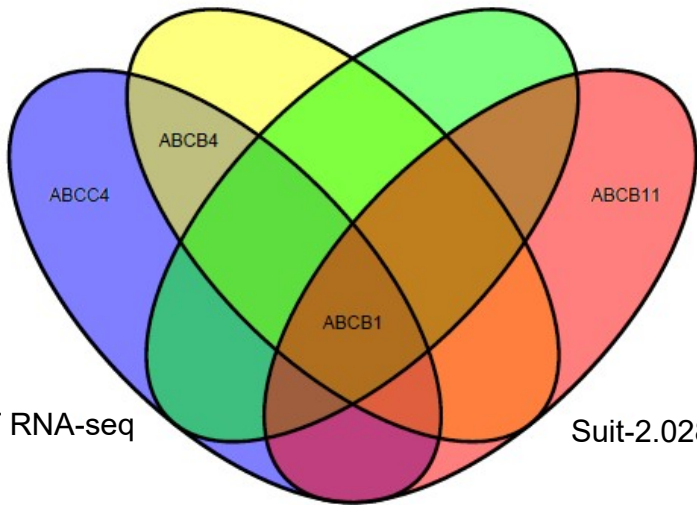

## Patu-T RNA-seq

## Suit-2.028 Prot

Fig. S3

Supplement: Supplementary file 8 — Additional file 8: Supplementary Fig. S3. Upregulation of ABC transporters in PR cells. Venn diagram showing upregulated ABC transporters in PR models. [file 13046_2023_2879_MOESM8_ESM.pdf]

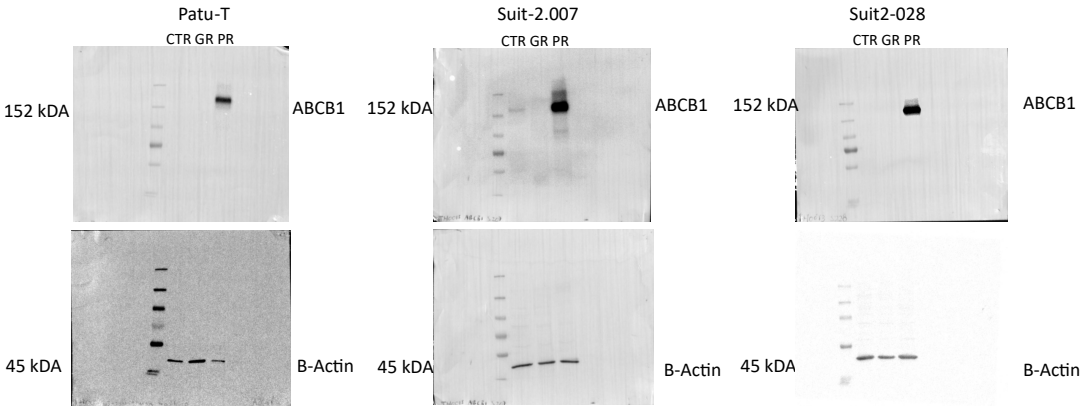

Fig. S4

Supplement: Supplementary file 9 — Additional file 9: Supplementary Fig. S4. Uncropped Western blot membranes for Patu-T, Suit-2.028 and Suit-2.007 CTR, PR, and GR cells stained for ABCB1 and B-actin. One biological replicate of western blot is shown for each cell line. Each sample was collected from untreated cells. [file 13046_2023_2879_MOESM9_ESM.pdf]

**A**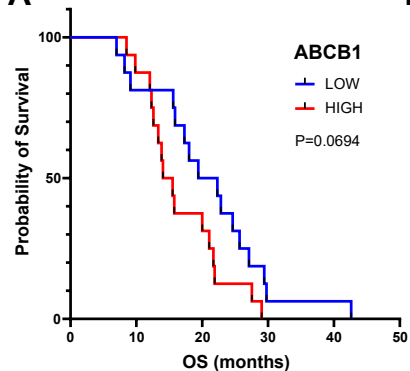**B**

|                         | No, %   | OS   | Logrank P |
|-------------------------|---------|------|-----------|
| <b>Age, years</b>       |         |      |           |
| ≤65                     | 10 (31) | 19.9 | 0.707     |
| >65                     | 22 (69) | 18.1 |           |
| <b>Sex</b>              |         |      |           |
| Male                    | 19 (59) | 20.6 | 0.313     |
| Female                  | 13 (41) | 16.2 |           |
| <b>Grading</b>          |         |      |           |
| 1-2                     | 18 (56) | 20.6 | 0.243     |
| 3                       | 14 (44) | 16.5 |           |
| <b>ABCB1 expression</b> |         |      |           |
| low                     | 15 (47) | 20.8 | 0.0694    |
| high                    | 17 (53) | 14.7 |           |

Fig. S5

Supplement: Supplementary file 10 — Additional file 10: Supplementary Fig. S5. ABCB1 upregulation correlates with poor survival. A. Kaplan-Meier curves of the patients that underwent surgery, grouped according ABCB1 expression, showing a trend towards reduced probability of survival in case of high (red) vs. low (blue) expression. B. Clinicopathological characteristics and correlation with mean overall survival (OS) of the PDAC patients. [file 13046_2023_2879_MOESM10_ESM.pdf]

**A****Suit-2.007 PR**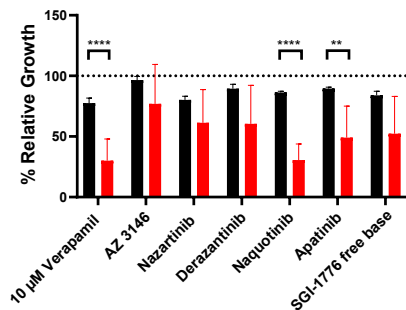**Suit-2.028 PR**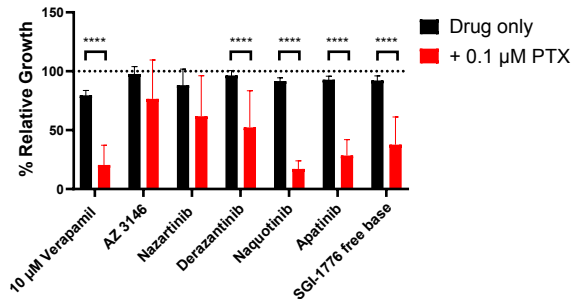**B****Suit-2.007 PR**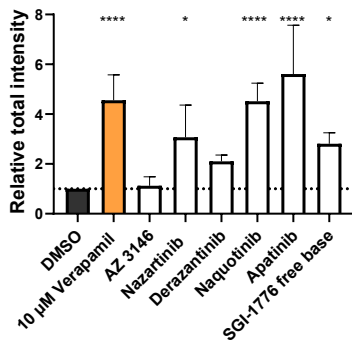**Suit-2.028 PR**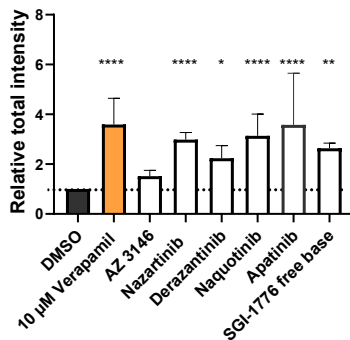

Fig. S7

Supplement: Supplementary file 12 — Additional file 12: Supplementary Fig. S7. KI screen validation in Suit-2.007 and Suit-2.028 PR cells. A. Impact on cell proliferation. Selected KIs from KI library screen were tested at 1 µM in absence (black bars) or presence (red bars) of 0.1 µM paclitaxel (PTX). Proliferation was assessed after 72 hours of treatment. Dotted line represents the DMSO control (100%). Experiments were performed in technical duplicates. Mean and SD of three independent experiments is shown. Ordinary one-way ANOVA was performed, followed by Šídák’s multiple comparisons test. **,p < 0.005; ****, p < 0.0001. B. Impact on Hoechst exclusion. Selected KIs from KI library screen were tested for their ability to prevent Hoechst exclusion in PR cells. Relative Hoechst signal intensity for the indicated treatments versus DMSO is shown. Mean and SD of 3 independent experiments performed in triplicates is shown. Ordinary one-way ANOVA with Dunnet’s post hoc test was used. *, p < 0.05; **, p < 0.005; ****, p < 0.0001. [file 13046_2023_2879_MOESM12_ESM.pdf]

### Patu-T

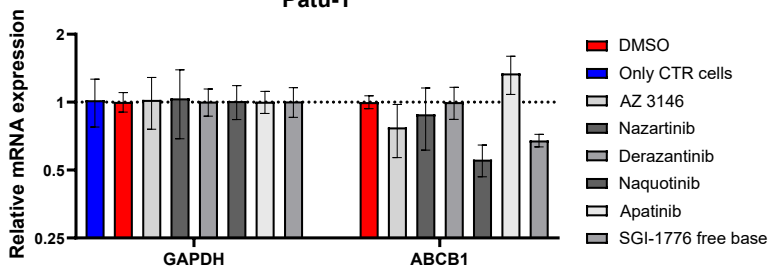

### Suit-2.007

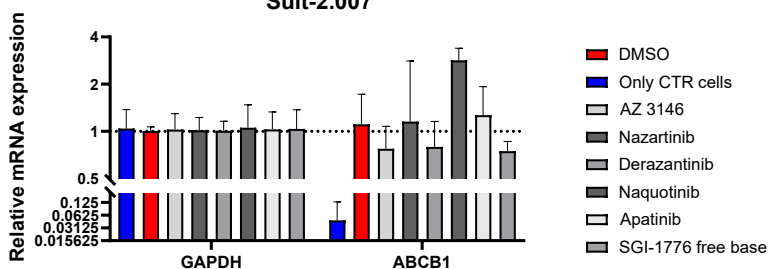

### Suit-2.028

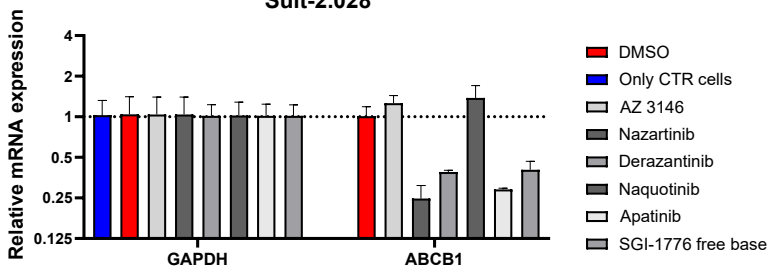

Supplement: Supplementary file 13 — Additional file 13: Supplementary Fig. S8. KI treatment of PR cells does not decrease ABCB1 expression to level of CTR cells. Gene expression of ABCB1 and GAPDH in PR cells after 48h of treatment with 1 µM of the indicated KI, measured by RT-qPCR and calculated as fold change (2-ΔΔCt compared to the DMSO control). Untreated CTR sample was included as negative control for ABCB1 expression (blue). Bars, mean of triplicates. 1 experiment was performed. [file 13046_2023_2879_MOESM13_ESM.pdf]
