# Supplementary material for: ABCB1 overexpression through locus amplification represents an actionable target to combat paclitaxel resistance in pancreatic cancer cells
Source: J Exp Clin Cancer Res. 2024 Jan 2;43:4. doi: 10.1186/s13046-023-02879-8 (PMC10759666; doi:10.1186/s13046-023-02879-8)
Supplement: Supplementary file 11 — Additional file 11: Supplementary Fig. S6. Verapamil mono treatment does not affect PR cell proliferation and verapamil does not sensitize to gemcitabine. A. Representative growth curves of 3 PR cell lines (red) exposed to verapamil concentration ranges, relative to DMSO control. Mean and SD of triplicates is shown. The experiment was repeated 3 times. B. Representative growth curves showing effect of 5 µM or 10 µM Verapamil on sensitivity to increasing concentrations of gemcitabine for CTR (left) or GR (right) PDAC cells. Mean and SD of triplicates is shown. The experiment was repeated 2 times. [file 13046_2023_2879_MOESM11_ESM.pdf]

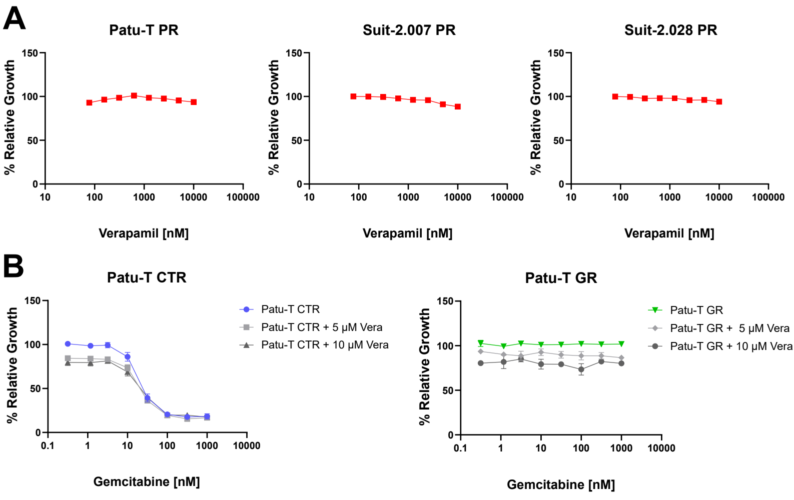
A
Patu-T PR
Suit-2.007 PR
Suit-2.028 PR
% Relative Growth
150
100
50
0
10
100
1000
10000
100000
Verapamil [nM]
B
Patu-T CTR
Patu-T GR
Patu-T CTR
Patu-T CTR + 5 μM Vera
Patu-T CTR + 10 μM Vera
Patu-T GR
Patu-T GR + 5 μM Vera
Patu-T GR + 10 μM Vera
0.1
1
Gemcitabine [nM]

Fig. S6
